# Emergency Department-based Intensive Care Unit Use Peaks Near Emergency Department Shift Turnover

**DOI:** 10.5811/westjem.2020.4.46000

**Published:** 2020-07-06

**Authors:** Nathan L. Haas, Henrique A. Puls, Andrew J. Adan, Colman Hatton, John R. Joseph, Christopher Hebert, David Hackenson, Kyle J. Gunnerson, Benjamin S. Bassin

**Affiliations:** * Michigan Medicine, Department of Emergency Medicine, Ann Arbor, Michigan; † Michigan Medicine, Division of Emergency Critical Care, Ann Arbor, Michigan; ‡ University of Cincinnati, Department of Emergency Medicine, Cincinnati, Ohio; § University of Washington, Department of Medicine, Division of Pulmonary, Critical Care and Sleep Medicine, Seattle, Washington; ¶ Michigan Medicine, Department of Internal Medicine, Ann Arbor, Michigan; || Michigan Medicine, Department of Anesthesiology/Critical Care, Ann Arbor, Michigan

## Abstract

**Introduction:**

The Emergency Critical Care Center (EC3) is an emergency department-based intensive care unit (ED-ICU) designed to improve timely access to critical care for ED patients. ED patients requiring intensive care are initially evaluated and managed in the main ED prior to transfer to a separate group of ED-ICU clinicians. The timing of patient transfers to the ED-ICU may decrease the number of handoffs between main ED teams and have an impact on both patient outcomes and optimal provider staffing models, but has not previously been studied. We aimed to analyze patterns of transfer to the ED-ICU and the relationship with shift turnover times in the main ED. We hypothesized that the number of transfers to the ED-ICU increases near main ED shift turnover times.

**Methods:**

An electronic health record search identified all patients managed in the ED and ED-ICU in 2016 and 2017. We analyzed the number of ED arrivals per hour, the number of ED-ICU consults per hour, the time interval from ED arrival to ED-ICU consult, the distribution throughout the day, and the relationship with shift turnover times in the main ED.

**Results:**

A total of 160,198 ED visits were queried, of which 5308 (3.3%) were managed in the ED-ICU. ED shift turnover times were 7 am, 3 pm, and 11 pm. The mean number of ED-ICU consults placed per hour was 221 (85 standard deviation), with relative maximums occurring near ED turnover times: 10:31 pm–11:30 pm (372) and 2:31 pm–3:30 pm (365). The minimum was placed between 7:31 am – 8:30 am (88), shortly after the morning ED turnover time. The median interval from ED arrival time to ED-ICU consult order was 161 minutes (range 6–1,434; interquartile range 144–174). Relative minimums were observed for patients arriving shortly prior to ED turnover times: 4:31 am – 5:30 am (120 minutes [min]), 12:31 pm – 1:30 pm (145 min), and 9:31 pm – 10:30 pm (135 min). Relative maximums were observed for patients arriving shortly after ED turnover times: 7:31 am – 8:30 am (177 min), 4:31 pm – 5:30 pm (218 min), and 11:31 pm – 12:30 am (179 min).

**Conclusion:**

ED-ICU utilization was highest near ED shift turnover times, and utilization was dissimilar to overall ED arrival patterns. Patients arriving immediately prior to ED shift turnover received earlier consults to the ED-ICU, suggesting these patients may have been preferentially transferred to the ED-ICU rather than signed out to the next team of emergency clinicians. These findings may guide operational planning, staffing models, and timing of shift turnover for other institutions implementing ED-ICUs. Future studies could investigate whether an ED-ICU model improves critically ill patients’ outcomes by minimizing ED provider handoffs.

## INTRODUCTION

From 2001–2009, the annual hours of critical care delivered in United States (US) emergency departments (ED) increased substantially, driven by an increasing proportion of ED visits requiring critical care and an increasing ED length of stay (LOS) for these patients.^1^ Concurrently, 33% of US intensive care unit (ICU) admissions from the ED have an ED LOS longer than six hours.^1^ This amount of ED boarding time of critically ill patients has been associated with increased hospital LOS, ICU LOS, morbidity, and mortality.^2^–^8^ Novel strategies are being investigated and implemented to combat this issue, including ED-based ICUs.^9^–^11^

In 2015 Michigan Medicine opened an ED-ICU, the Joyce and Don Massey Family Foundation Emergency Critical Care Center (EC3), with the objective of improving timely access to critical care for patients in the ED.^11^ It contains five resuscitation bays and nine patient rooms immediately adjacent to the main ED. All ED patients are initially evaluated and treated by the main ED team in resuscitation bays or ED treatment rooms, and are subsequently transitioned to the EC3 team for ongoing critical care delivery.

Transitions of care in the ED occur at the end of every shift. They are used to hand off important information from clinician to clinician and are crucial for the continuity of patient care. However, breakdown in communication during transitions of care is a leading root cause of sentinel events,^12^ and is associated with delays of care, near misses, and ICU transfers.^13^, ^14^ With the increase in hours of critical care delivery in US EDs,^1^ transitioning care of critically ill ED patients can prove a complex task susceptible to high error rates with serious consequences.^15^ One factor to consider when implementing an ED-ICU is its impact on transitions of care within the ED.

The timing of patient transfers to the ED-ICU may decrease the number of transitions of care between ED teams and have an impact on both patient outcomes and optimal provider staffing models, but has not been previously studied. Investigating methods to smooth and load level transitions of care from the ED to an ED-ICU may provide insight into more effective resource utilization, staffing models, and patient throughput. The objective of this study was to examine patterns of consultation to the ED-ICU and their relationship with shift turnover times in the main ED. We hypothesized that the number of transfers to the ED-ICU increases near ED shift turnover times.

## METHODS

This was a retrospective review of data from all ED visits from January 1, 2016–December 31, 2017. This study was conducted at a single, large, academic medical center with approximately 75,000 adult ED visits per year. Data collection and analysis were performed in 2018, and manuscript preparation was conducted in 2019. The institutional review board (IRB) at the University of Michigan reviewed and approved this study (HUM00171720) and granted exemption from continuing IRB review. We treated all data in a manner compliant with the Security Rule and the Privacy Rule of the Health Insurance Portability and Accountability Act. This study is reported in compliance with the Strengthening the Reporting of Observational Studies in Epidemiology (STROBE) guidelines.^16^

**Population Health Research Capsule**

What do we already know about this issue?

*The timing of consultations to an ED-ICU may decrease handoffs between main ED teams and have an impact on outcomes and optimal staffing models*.

What was the research question?

Do patterns of consultation to an ED-ICU correspond to ED shift turnover times?

What was the major finding of the study?

*ED-ICU utilization was highest near ED shift turnover times and dissimilar to overall ED arrival patterns*.

How does this improve population health?

*These findings may guide operational planning, staffing models, and timing of shift turnover for other institutions implementing ED-ICUs*.

We identified all adult (>18 years) patients presenting to the adult ED in 2016 or 2017 via a search of electronic health records (EHR), and all patients managed in the ED-ICU were identified and included for analysis. De-identified patient data, including ED arrival time, ED-ICU consult order time, time changed to ED-ICU status, and reason for ED-ICU consult were queried and analyzed. We divided patients into 24 cohorts based on the hour of day of ED arrival time.

The methods used in this study minimized several types of bias associated with retrospective studies. We obtained data from all patients presenting to the ED with a consult to the ED-ICU, thus minimizing selection bias. The operational data used for the analyses performed was for all patients included and are a result of regular workflows in the ED, therefore unlikely to be subject to inaccuracies and minimizing both information bias and measurement errors. The study size was arrived at by defining the time interval of patient presentations to include the following: the ED-ICU opened in 2015, and we allowed for a “wash-out period” prior to collecting and analyzing all patient encounters in 2016 and 2017.

## RESULTS

**Figure 1:**
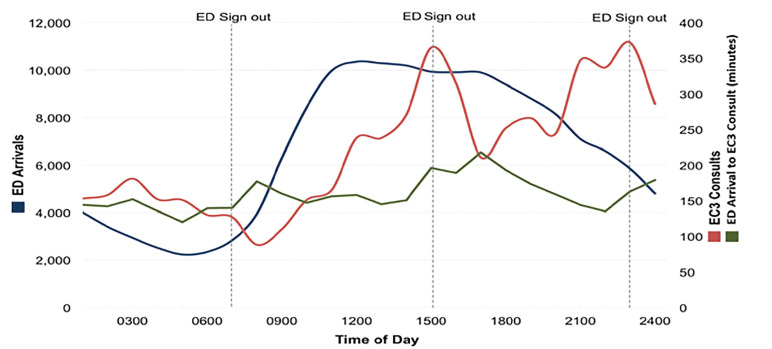
Comparison between ED arrivals (blue), ED-ICU (EC3) consults (red), and time from ED arrival to ED-ICU consult (green). *ED*, emergency department; *ED-ICU*, emergency department-based intensive care unit; *EC3*, emergency critical care center.

We identified and analyzed a total of 160,198 ED patient encounters, of which 5308 (3.3%) had ED-ICU consults placed. ED-ICU consult reasons included severe sepsis/septic shock (15%); altered mental status/overdose (10%); metabolic, including diabetic ketoacidosis/electrolytes (9%); gastrointestinal bleed (7%); respiratory distress/respiratory failure (5%); and other (41%). ED shift turnover times were 7 am, 3 pm, and 11 pm, whereas ED-ICU shift turnover times were 8 am and 8 pm. The main results of the study are summarized in Figure 1.

The overall rate of ED arrivals per hour for the study period was 9.13. The number of ED arrivals per hour was maximum at 11:31 am – 12:30 pm (10,353 total ED arrivals), remained relatively stable until 4:31 pm–5:30 pm, and steadily decreased until a minimum at 4:31 am – 5:30 am (2234 total ED arrivals). The overall rate of ED-ICU consults placed per hour for the study period was 0.30. We identified two relative maximums in the number of ED-ICU consults per hour, both occurring at ED shift turnover times: 10:31 pm – 11:30 pm (372) and 2:31 pm – 3:30 pm (365). The minimum number of ED-ICU consults per hour occurred between 7:31 am – 8:30 am (88), shortly after the 7 am shift turnover time. Two additional relative minimums in ED-ICU consults per hour were observed shortly after the 3 pm and 11 pm sign-out times.

During day hours (8:31 am–8:30 pm), there were 111,640 ED arrivals and 2826 ED-ICU consults (2.5%), while during night hours (8:31 pm–08:30 am ) there were 48,558 ED arrivals and 2482 ED-ICU consults (5.1%). The median interval from ED arrival time to ED-ICU consult order was 161 minutes (range 6–1434; interquartile range 144–174). Relative minimums were observed for patients arriving shortly prior to ED shift turnover times: 4:31 am – 5:30 am (120 minutes), 12:31 pm–1:30 pm (145 minutes), and 9:31 pm – 10:30 pm (135 minutes). Relative maximums were observed for patients arriving shortly after ED shift turnover times: 7:31am – 8:30 am (177 minutes), 4:31 pm – 5:30 pm (218 minutes), and 11:31 pm–12:30 am (179 minutes).

## DISCUSSION

Results of this study indicate that ED-ICU utilization was highest near ED shift turnover times, and ED-ICU utilization was dissimilar to overall ED arrival patterns. Patients arriving immediately prior to ED shift turnover received earlier consults to the ED-ICU, suggesting these patients may have been preferentially transferred to the ED-ICU rather than handed off to the next team of ED providers. These findings may guide operational planning, staffing models, and timing of shift turnover for other institutions implementing ED-ICUs.

As shift end approaches, off-going emergency clinicians must determine disposition or transition the care of each patient being managed to the next emergency clinician. Breakdown in communication during transitions of care is a leading root cause of sentinel events,^12^ and is associated with delays of care, near misses, and ICU transfers.^13^,^14^ We hypothesized that emergency clinicians would opt to consult an ED-ICU for critically ill ED patients near the end of their shifts rather than transition care to the oncoming emergency clinician team to potentially mitigate these breakdowns in communication. We hypothesized maximum numbers of ED-ICU consults near shift turnover times (7 am, 3 pm, 11 pm) and quicker ED-ICU consults for patients arriving shortly prior to (compared to shortly after) ED shift turnover times would be observed. The results of this study support both hypotheses. We observed relative maximums in consults to an ED-ICU near ED shift turnover times, occurring independently of ED volume, as evidenced by the relative maximum number of ED-ICU consults near the 11 pm ED shift turnover despite declining total ED volume (Figure 1).

The retrospective nature of the data gives strength to our findings because the clinician decisions were not influenced by the fact their behavior was being observed. It is feasible that when faced with the option of transferring a critically ill patient to an ED-ICU or transitioning care to an oncoming ED team, emergency physicians opt for a shorter ED LOS and more rapid transfer to an ED-ICU near the end of their shifts.

A relative minimum time to ED-ICU consult was observed for patients arriving shortly before shift turnover, suggesting clinicians opt to offload critically ill patients rather than transition care to an oncoming physician. Simultaneously, emergency physicians appear to preferentially manage critically ill patients for longer durations when more time is available during their shifts. A relative maximum time to ED-ICU consult was observed for patients arriving just after shift turnover, suggesting clinicians were more likely to perform further resuscitative actions early in their shifts.

Future studies could investigate whether an ED-ICU model improves critically ill patients’ outcomes by minimizing ED clinician handoffs and could also assess the external validity of these findings in other institutions.

## LIMITATIONS

A limitation of this study is the lack of patient-oriented outcomes, and we cannot imply better or worse outcomes related to the time of transfer to ED-ICU based on data collected for this study. We were unable to assess for confounding and recognize that additional factors could influence how quickly an emergency clinician decides to transfer a patient to the ED-ICU. These could include factors such as patient volume, acuity of additional patients being managed, availability of beds in the ED-ICU or inpatient ICUs, and whether the managing physician was an emergency medicine-intensivist. It is unknown whether the observed temporal trends of ED-ICU utilization are similar to other forms of ED disposition. The focus of this study was to evaluate patterns of consultations to the ED-ICU to better inform operational and staffing planning for institutions with or considering an ED-ICU. Therefore, we did not perform an analysis of consultation or disposition data to other units and did not explore whether they follow similar temporal characteristics. Our data generates the question of whether these temporal trends would also be observed with consultation and disposition to other services or units. This could be further explored in future research. This study was conducted at a single center in the United States, and external validity / generalizability of results is unknown.

## CONCLUSION

Utilization of an ED-ICU was highest near ED shift turnover times, and utilization was dissimilar to overall ED arrival patterns. Patients arriving immediately prior to ED shift turnover received earlier consults to the ED-ICU. A possible explanation for this observation is preferential transfer to the ED-ICU over transitioning care to the oncoming emergency clinician team. These findings may aid other institutions implementing ED-ICUs for operational planning, staffing models, and timing of shift turnovers. Future studies could investigate whether an ED-ICU model improves critically ill patients’ outcomes by minimizing ED provider handoffs.
